# Chemical Composition and *In Vitro* Cytotoxicity of Essential Oils from Leaves and Flowers of *Callistemon citrinus* from Western Himalayas

**DOI:** 10.1371/journal.pone.0133823

**Published:** 2015-08-26

**Authors:** Dharmesh Kumar, Mahesh Sukapaka, G. D. Kiran Babu, Yogendra Padwad

**Affiliations:** 1 Regulatory Research Center, Biotechnology Division, CSIR—Institute of Himalayan Bioresource Technology (CSIR-IHBT), Post Box No. 6, Palampur-176 061, Himachal Pradesh, India; 2 Natural Plant Products Division, CSIR—Institute of Himalayan Bioresource Technology (CSIR-IHBT), Post Box No. 6, Palampur-176 061, Himachal Pradesh, India; UMR INSERM U866, FRANCE

## Abstract

**Background:**

Plant-based traditional system of medicine continues to play an important role in healthcare. In order to find new potent source of bioactive molecules, we studied the cytotoxic activity of the essential oils from the flowers and leaves of *Callistemon citrinus*. This is the first report on anticancer potential of essential oils of *C*. *citrinus*.

**Methods:**

Cytotoxicity of essential oil was evaluated using sulfo-rhodamine B (SRB) assay against human lung carcinoma (A549), rat glioma (C-6), human colon cancer (Colo-205) and human cervical cancer (SiHa) cells. Apoptosis induction was evaluated by caspase-3/7 activity which was further confirmed by western blotting. Percentage cell apoptosis was determined by Annexin V based dead cell assay followed by DNA content as cell cycle analysis against A549 and C-6 cells. While 3-(4,5-dimethythiazol-2-yl)-2,5-diphenyl tetrazolium bromide (MTT) assay was used to check the toxicity against normal human peripheral blood mononuclear cells (PBMCs), the immunomodulatory activity on mouse splenocytes was evaluated using SRB assay.

**Results:**

The GC and GC-MS analysis of these essential oils revealed high content of *α*-pinene (32.3%), limonene (13.1%) and *α*-terpineol (14.6%) in leaf sample, whereas the flower oil was dominated by 1,8-cineole (36.6%) followed by *α*-pinene (29.7%). The leaf oil contained higher amount of monoterpene hydrocarbons (52.1%) and sesquiterpenoids (14%) as compared to flower oil (44.6% and 1.2%, respectively). However, the flower oil was predominant in oxygenated monoterpenes (43.5%). Although both leaf and flower oils showed highest cytotoxicity on A549 cells (61.4%±5.0 and 66.7%±2.2, respectively), only 100 μg/mL flower oil was significantly active against C-6 cells (69.1%±3.1). Interestingly, no toxicity was recorded on normal cells.

**Conclusion:**

Higher concentration of 1,8-cineole and/or synergistic effect of the overall composition were probably responsible for the efficacy of flower and leaf oils against the tested cells. These oils may form potential source of natural anti-cancer compounds and play important role in human health.

## Introduction

Bottlebrush [*Callistemon citrinus* (Curtis) Skeels syn.: *C*. *lanceolatus* D.C. and *Metrosideros citrina* Curtis, family Myrtaceae] is a woody aromatic ornamental plant that grows in different tropical and subtropical regions of the world [1,2]. The plants of the genus *Callistemon* have been reported to possess antithrombotic, anti-inflammatory and anti-staphylococcal activities. The plants also have nematocidal, larvicidal, pupicidal activities and are often used as insect repellent [3–9]. While *C*. *viminalisis* is used for treating hemorrhoids in Chinese traditional medicine [10], the methanolic and ethanolic leaf extracts of *C*. *citrinus* showed antibacterial and cardioprotective activities in rats [11,12]. The ethanolic extract of stem was also reported to show free radical scavenging activity and elastase inhibition [13]. Particularly, the bark extract has cytotoxicity against A549 cells [14]. The aerial parts of *C*. *citrinus* also find use in ethnic medicines [15].

The leaf oils of *C*. *citrinus* are known to have antimicrobial, fungitoxic, antinociceptive and anti-inflammatory activities [16,17]. Chemical investigations revealed that the flowers and leaves of the plant are rich in essential oils comprising of 1,8-cineole followed by *α-* and *β*-pinenes, *α-*terpineol, *α-*phellandrene, limonene, *α-*terpinene, linalool, *trans*-pinocarveol, terpinen-4-ol and geraniol [1,16,18–20]. Among these, the most widely distributed 1,8-cineole is known for its medicinal and flavouring properties [21,22]. It is also an environmental friendly compound with potential to replace Ozone Depleting Industrial Solvents (ODIS). This is important because ODIS are currently being phased out as per the Montreal Protocol [23].

In view of the above properties and bioactivity of *C*. *citrinus*, the aim of the present study was to evaluate the anticancer potential of the flower and leaf oils against human lung carcinoma (A549), rat glioma (C-6), human colon cancer (Colo-205) and human cervical cancer (SiHa) cells. The study also aimed at confirming the cytotoxicity by Annexin V based dead cell assay and caspase activation against A549 and C-6 cells.

## Material and Methods

### Ethics Statement

*C*. *citrinus* is neither an endangered nor a protected species in India. Lymphocyte proliferation assay protocol using human peripheral blood mononuclear cells (PBMCs) was approved by the Institutional Ethics Committee (IEC) of CSIR-Institute of Himalayan Bioresource Technology. Written consent as per the standard operating procedure was obtained from the volunteer(s) before collection of the blood samples. The protocol for isolation of splenocytes from mice was approved by Institutional Animal Ethical Committee (IAEC) of CSIR-Institute of Himalayan Bioresource Technology.

### Plant Material

**Fig 1:**
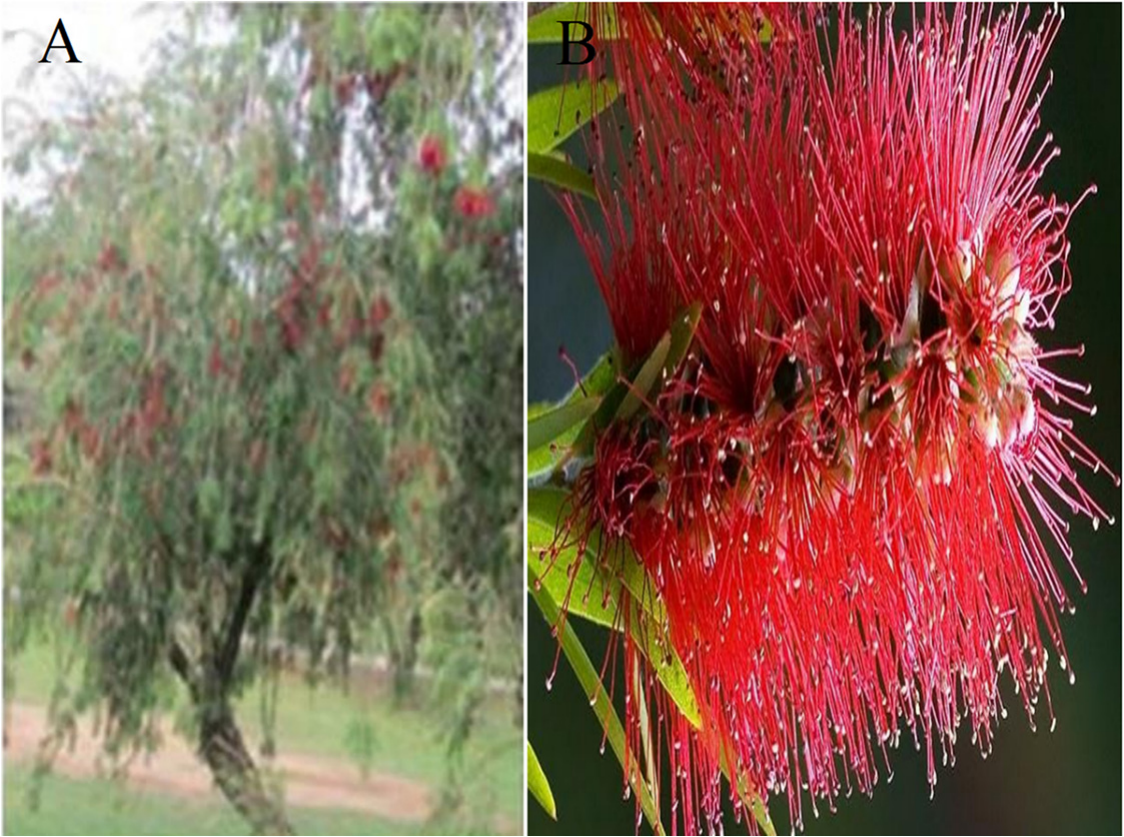
(A) *C*. *citrinus* plant. (B) Flower of *C*. *citrinus*.

Flowers and leaves of *C*. *citrinus* were collected in May, 2012 from CSIR-IHBT Palampur campus (altitude 1,300 m above the mean sea level) (Fig 1A and 1B).

### Hydrodistillation of *C*. *citrinus*

Hydrodistillation of *C*. *citrinus* leaves (4.0 kg fresh weight) and flowers (4.1 kg fresh weight) were carried out in a Clevenger-type apparatus. The hydrodistillation process was continued for 3.5 h after appearance of first drop of distillate. The oil samples collected were dried over anhydrous sodium sulfate, filtered and used for GC and GC-MS analysis.

### GC and GC-MS Analysis

GC analysis of essential oil samples was performed on Shimadzu GC-2010 equipped with flame ionization detector (FID) and DB-5MS Ultra Inert capillary column (30 m x 0.25 mm i.d., film thickness 0.25 μm, 5% phenyl methylpolysiloxane) using nitrogen as auxiliary carrier gas with flow rate of 4 mL/min. Oven temperature was programmed from 40 to 220°C at the rate of 4°C/min, held isothermally at 40°C and at 220°C for 4 and 15 min, respectively. 10 μL oil samples were mixed with 2 mL dichloromethane (DCM) and 2 μL of this solution was injected. Injector port and detector temperatures were kept at 220°C and 250°C, respectively. GC-MS analysis was done on Shimadzu QP2010 series fitted with AOC-20i auto-sampler and DB-5MS capillary column (30 m x 0.25 mm i.d., film thickness 0.25 μm). Helium (99.99% pure) was used as carrier gas with 1.28 mL/min flow rate, linear velocity 40.8 cm/s, pressure 69.3 kPa, split ratio 1:50, mass scan 50–800 amu at a sampling rate of 1.0 scan/s, scan speed: 1666 u/s, interval: 0.5 s. The oven temperature was programmed as mentioned for GC analysis. Electron impact ionization at 70 eV with 0.9 kV detector voltage was used. 10 μL oil samples were mixed with 2 mL DCM (HPLC grade) and 2 μL of this solution was injected. Ion source temperature was 200°C, interface temperature was 250°C, and injector temperature was maintained at 250°C. The constituents were identified with the help of relative retention indices and by comparison with known mass spectral data [24,25], National Institute of Standards and Technology (NIST) [26] and our own libraries. A mixture of *n*-alkanes (C_8_-C_32_) was used as reference for the calculation of relative retention indices (RRI) in temperature-programmed run.

### Cell lines and cell culture

Human lung carcinoma (A549), human cervical cancer (SiHa), human colon cancer (Colo-205) and rat glioma (C-6) cells were obtained from National Centre for Cell Science (NCCS), Pune. A549 cells were grown in Ham’s Nutrient Mixtures F-12 medium (Invitrogen Biosciences, India), SiHa and Colo-205 cells were cultured in Roswell Park Memorial Institute-1640 [(RPMI-1640) (Invitrogen Biosciences, India)] and C-6 cells in Dulbecco's Modified Eagle Medium [(DMEM) (Invitrogen Biosciences, India)], supplemented with 10% heat-inactivated fetal bovine serum (Invitrogen Biosciences, India) and 1% antibiotic antimycotic solution (Ref. No. 15240–062, Invitrogen Biosciences, India). The cells were maintained at 37°C in 5% CO_2_ humidified atmosphere [27,28].

### Isolation of human PBMCs

PBMCs were isolated from freshly collected peripheral venous blood drawn in heparinized tubes. The blood sample was diluted 1:1 with 1M phosphate buffered saline (PBS). The cells were isolated by Ficoll density gradient centrifugation [29]. In brief, 4 mL of Ficoll–HiSep (Hi-Media, India) was taken in a 15 mL tube and 10 mL of diluted blood was loaded without disturbing the interface and centrifuged at 1800 x g for 30 min. PBMC ring was separated and washed thrice with PBS by centrifugation at 200 x g for 10 min. The cells were suspended in RPMI-1640 medium containing 10% heat-inactivated fetal bovine serum, 2 mM glutamine, 110 mg/mL sodium pyruvate and 1% antibiotic antimycotic.

### SRB assay

The cells were trypsinized and washed twice with PBS by centrifugation and incubated at a density of 2x10^4^ cells/well (A549, SiHa, Colo-205 and C-6) in 96 well plates in 100 μL complete medium. Several concentrations (10, 20, 50 and 100 μg/mL) of flower and leaf oils of *C*. *citrinus* in 100 μL complete medium were added. Vinblastine (1 μM) was used as positive control, whereas cells alone supplemented with complete medium were used as negative control. Plates were incubated at 37°C for 48 h in CO_2_ incubator. After 48 h, 50 μL 50% trichloroacetic acid was added to the wells and the plates were kept at 4°C for 1 h. The plates were flicked and washed five times with water and then air-dried. Subsequently, 100 μL SRB solution was added and incubated for 30 min at room temperature. After incubation, plates were washed six times with 1% acetic acid, air dried and 10 mM tris base (Sigma Aldrich, India), was added. The absorbance was measured using microplate reader (BioTeK Synergy H1 Hybrid Reader) at 540 nm [27].

### Morphological changes

The morphological changes in A549 and C-6 cells treated with both flower and leaf oils for 24 and 48 h were observed and images were captured using fluorescent microscope (Nikon Eclipse T_i_-S) at 10X [27].

### MTT assay

PBMCs were seeded at a density of 3x10^5^ cells per well in 100 μL of complete RPMI-1640 medium. The assay was performed using 96 well plates (Corning Costar, USA) at 37°C with 5% CO_2_, for 72 h. The cytotoxicity of flower and leaf oils on mononuclear cells (PBMCs) at 10, 20, 50 and 100 μg/mL was evaluated [30]. The cell survival was identified by MTT assay and the color intensity was quantified by absorbance measurement at 570 nm [31].

### Splenocyte assay

Spleen was excised from mice and transferred to 5 mL complete RPMI-1640 medium. Tissue was macerated using syringe plunger and centrifuged at 450 x g for 10 min. The pellet was resuspended in 2 mL erythrolysis buffer and incubated for 5 min at 37°C. After incubation the cells were washed twice by centrifugation at 450 x g for 10 min. The pellet was again suspended in 5 mL of complete RPMI-1640 medium and viable cells were counted using haemocytometer. Cytotoxicity of flower and leaf oils was evaluated against splenocytes at a density of 1x10^5^ cells/well in 96 well plates at four different concentrations (10, 20, 50 and 100 μg/mL) by SRB assay for 24, 48 and 72 h [32].

### Caspase-3/7 activity

A549 and C-6 cells were plated in 96 well plates at a density of 2*×*10^3^ cells/well in 50 μL complete medium. Four different concentrations (10, 20, 50 and 100 μg/mL) of both flower and leaf oils in 50 μL complete medium were added to the wells. The plates were incubated at 37°C for 8 to 12 h in CO_2_ incubator. After incubation, 100 μL caspase-3/7 reagent was added followed by incubation at room temperature for 2 to 2.5 h. Total caspase activity was detected using Apo-ONE homogeneous caspase-3/7 assay kit (Promega). The caspase-3/7 activity was calculated by measuring the net Relative Fluorescence Units (RFU) using microplate reader at an emission and excitation wavelengths of 530 and 485 nm, respectively [27].

### Apoptosis Assay

A549 and C-6 cells were plated in 12 well plates at a density of 5×10^5^ cells/well and were treated with flower and leaf oils at different concentrations (10, 20, 50 and 100 μg/mL) for 12 and 24 h, respectively. The percent cell apoptosis was determined according to the supplier’s instructions provided with the kit (Millipore, Catalogue No. MCH100105). Adherent and floating cells were collected, washed with PBS, resuspended in 100 μL complete medium and then stained with 100 μL Annexin V and dead cell detection reagent. Further, the cells were incubated for 20 min at room temperature and apoptosis was measured immediately using Muse Cell Analyzer (Millipore) [33].

### Western blotting

Blotting was performed as described earlier [34]. In brief, the cells were harvested and lysed in RIPA buffer (Sigma-Aldrich). Cell lysates stored at -80°C until analysis. An amount of 20 μg proteins per sample were separated by 10% SDS-polyacrylamide gel electrophoresis and transferred to PVDF membrane (Whatman Westran S). The membranes were blocked for 1 h in Western Blocker Solution (Sigma-Aldrich). Immunoblots were probed with rabbit anti-human caspase-3, 7, 9 and PARP monoclonal antibody (1:1000; Cell Signaling Technologies, USA) Whereas for CDKs studies CDK-2, 6, 7, 9 and CDC-2 monoclonal antibody (1:1000; Cell Signaling Technologies, USA) were used. Blots were allowed to incubate for 3 h with primary antibodies followed by horseradish peroxidase-linked anti-rabbit and anti-mouse IgG whole antibody (1:5,000; Cell Signaling Technologies, USA) for 1 h. All washing steps were performed using 0.05% PBST and all incubations were performed at room temperature. After incubation, the protein bands were visualized using ECL western blotting substrate (Thermo Scientific, Rockford, USA). All membranes were stripped and probed with anti β-tubulin antibody (Santacruz, USA) to use as loading control.

### Cell cycle analysis

A549 and C-6 cells were plated in 6-well plates at a density of 1x10^6^ cells/well. After 24 h treatment with 20, 50, and 100 μg/mL *C*. *citrinus* flower and leaf oils, the DNA content was determined. The cell cycle analysis was performed according to the supplier’s instructions provided with the kit (Millipore, Catalogue No. MCH100106). Briefly, cells were harvested after treatment, washed with PBS by centrifugation at 300 x g for 5 min, fixed with 70% (v/v) ice-cold ethanol and kept at -20°C for 12 h. Finally, the cells were washed with PBS and stained with 200 μL cell cycle reagent. The cell suspension was incubated in dark at room temperature for 30 min and the DNA content was determined using Muse Cell Analyzer (Millipore) [35].

### Data analysis

Data represents the results of 3 independent experiments. Standard deviation was calculated using Microsoft Excel. Whereas, the *p* value was calculated with the help of GraphPad QuickCalcs: *t test* calculator available freely online (http://www.graphpad.com/quickcalcs/ttest1/?Format=SD). All data are presented as a mean value with its standard deviation indicated (mean ± SD).

## Results

### Essential oil yields and chemical composition

The leaves and flowers of *C*. *citrinus* yielded 0.54% and 0.12% of essential oils, respectively. Nineteen in leaf oil and seventeen components in flower oil were identified by GC-FID accounting for 90.9% and 91.9% of total oil, respectively (Table 1). As compared to flowers, the leaf oil contained higher concentration of *α*-pinene (32.3%), limonene (13.1%), *α*-terpineol (14.6%), spathulenol (2.5%), *allo*-aromadendrene (2.4%), viridiflorol (2.1%), *β*-caryophyllene and aromadendrene (1.4% each). However, the flower oil contained 36.6% 1,8-cineole, 29.7% *α*-pinene, 5.6% *α*-terpineol, 4.4% *α*-phellandrene, 4% limonene and 2.9% *α*-terpinene.

**Table 1:** Chemical composition (%) of flower and leaf oils produced by hydrodistillation from Callistemon citrinus.

Compound	RRI^1^	Flower	Leaf oil
		oil	
Isoamyl acetate	884	1.0	—-^2^
Isobutyl isobutyrate	912	1.1	—-
*α*-Thujene	921	0.8	—-
*α*-Pinene	928	29.7	32.3
*β*-Pinene	971	0.6	1.9
Myrcene	987	—-	1.0
*α*-Phellandrene	1002	4.4	2.1
*α*-Terpinene	1013	2.9	—-
*p*-Cymene	1022	1.0	0.7
Limonene	1026	4.0	13.1
1,8-Cineole	1028	36.6	9.8
*γ*-Terpinene	1055	0.4	1.0
*α*-Terpinolene	1083	0.8	—-
Linalool	1106	0.8	—-
Terpinen-4-ol	1183	0.5	0.6
*α*-Terpineol	1202	5.6	14.6
*β*-Caryophyllene	1417	0.4	1.4
Aromadendrene	1437	—-	1.4
*allo*-Aromadendrene	1458	—-	2.4
Ledene	1490	0.8	1.6
*β*-Sesquiphellandrene	1518	—-	0.9
Ledol	1573	—-	0.9
Spathulenol	1584	—-	2.5
Viridiflorol	1592	—-	2.1
Globulol	1612	—-	0.8
*β*-Asarone	1624	0.5	—-
**Total**		**91.9**	**91.1**
Linear compounds (LC)		2.1	—-
Monoterpene hydrocarbons (MH)	44.6	52.1	
Oxygenated monoterpenes (OM)	43.5	25.0	
Sesquiterpene hydrocarbons (SH)	1.2	7.7	
Oxygenated sesquiterpenes (OS)	—-	6.3	
Phenyl propanoids (PP)		0.5	—-
Oil yields (v/w %)		0.12	0.54

^1^ RRI, Relative Retention Indices on DB-5MS column were calculated from retention times relative to *n*-alkanes

^2^ Absent

### Cytotoxicity- Anticancer activity

**Fig 2:**
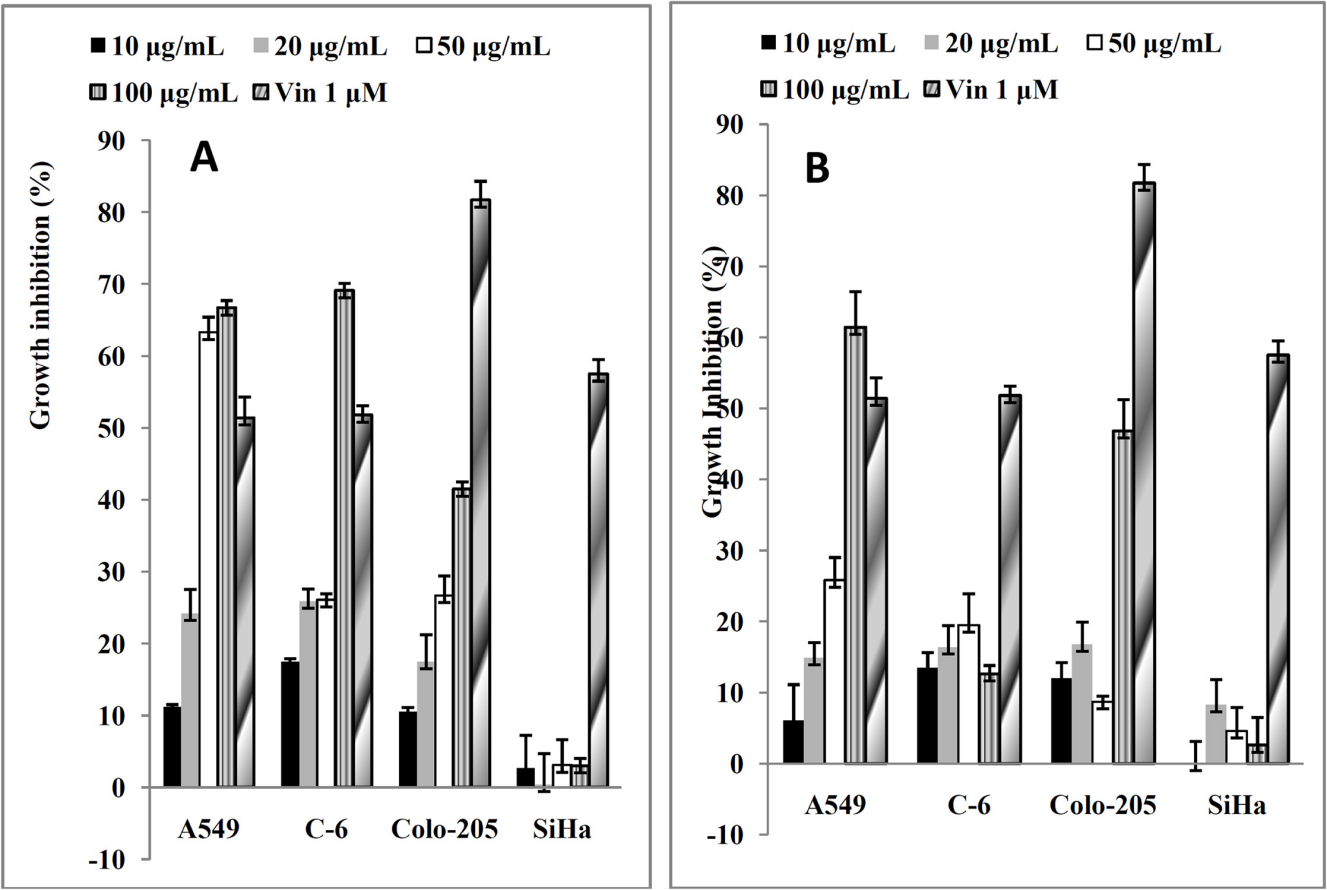
*In vitro* cytotoxicity of *C*. *citrinus* oil against A549, C-6, Colo-205 and SiHa cells by SRB assay. (A) Flower oil. (B) Leaf oil.

**Fig 3:**
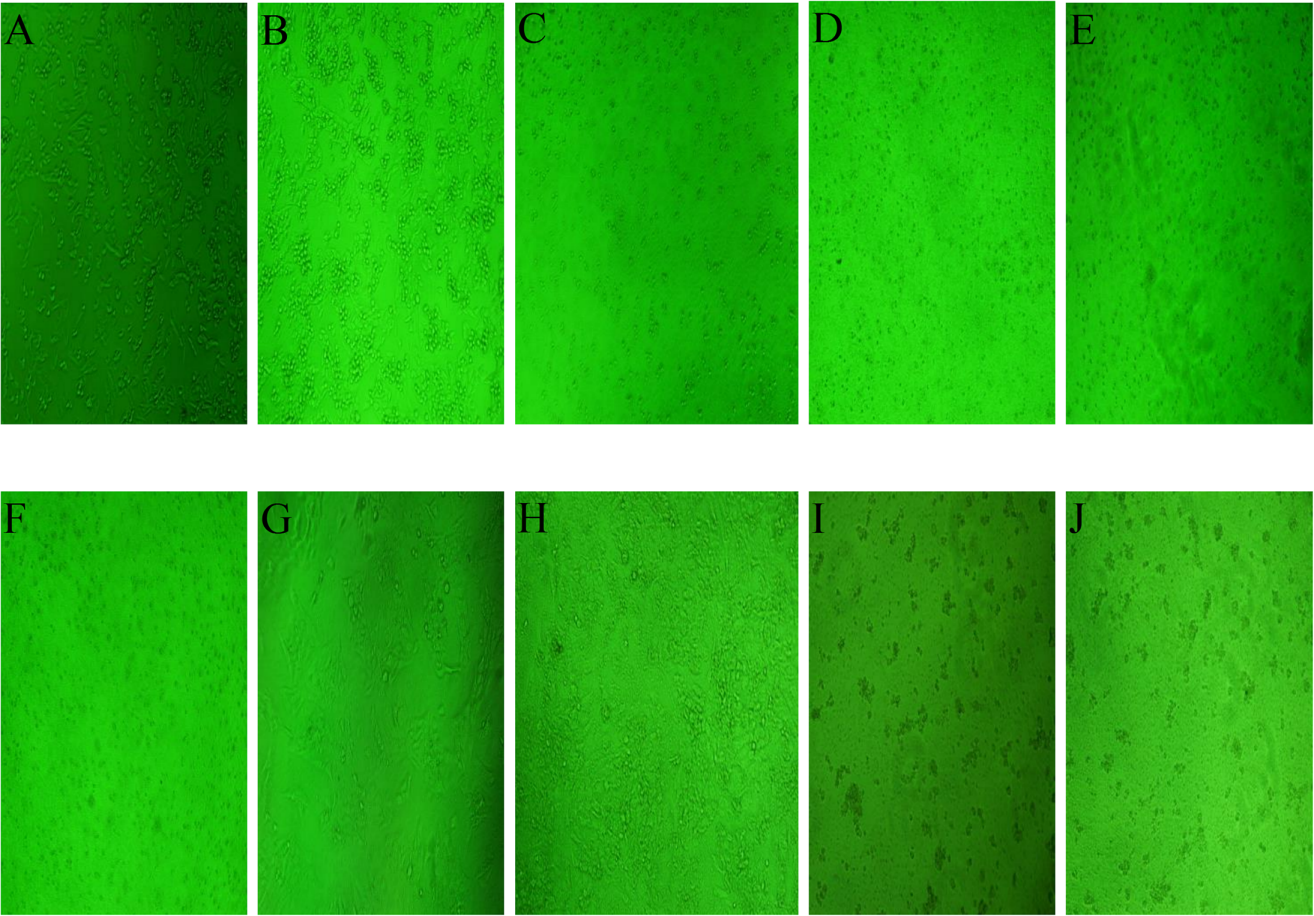
Microscopic images at 10X of A549 (A-F) and C-6 (G-J) cells treated for 24–48 h with 100 μg/mL of leaf and flower oils of C. citrinus (A) Control, no treatment after 24 h. (B) Control, no treatment after 48 h. (C) After 24 h treatment with flower oil. (D) After 48 h treatment with flower oil. (E) After 24 h treatment leaf oil. (F) After 48 h treatment with leaf oil. (G) Control, no treatment after 24 h. (H) Control, no treatment after 48 h. (I) After 24 h treatment with flower oil. (J) After 48 h of treatment with flower oil.

**Fig 4:**
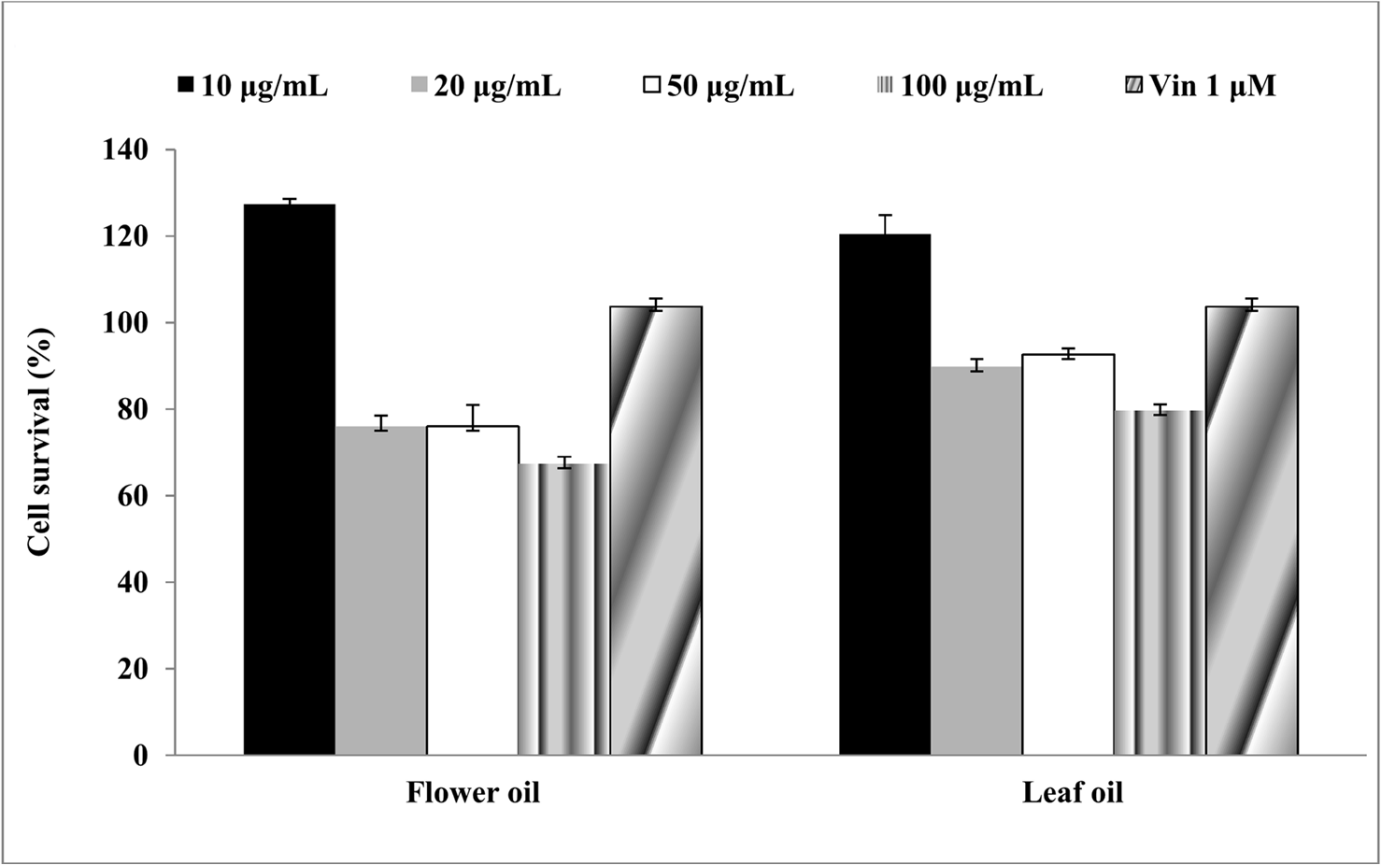
*In vitro* cytotoxicity of leaf and flower oils against human PBMCs by MTT assay.

**Fig 5:**
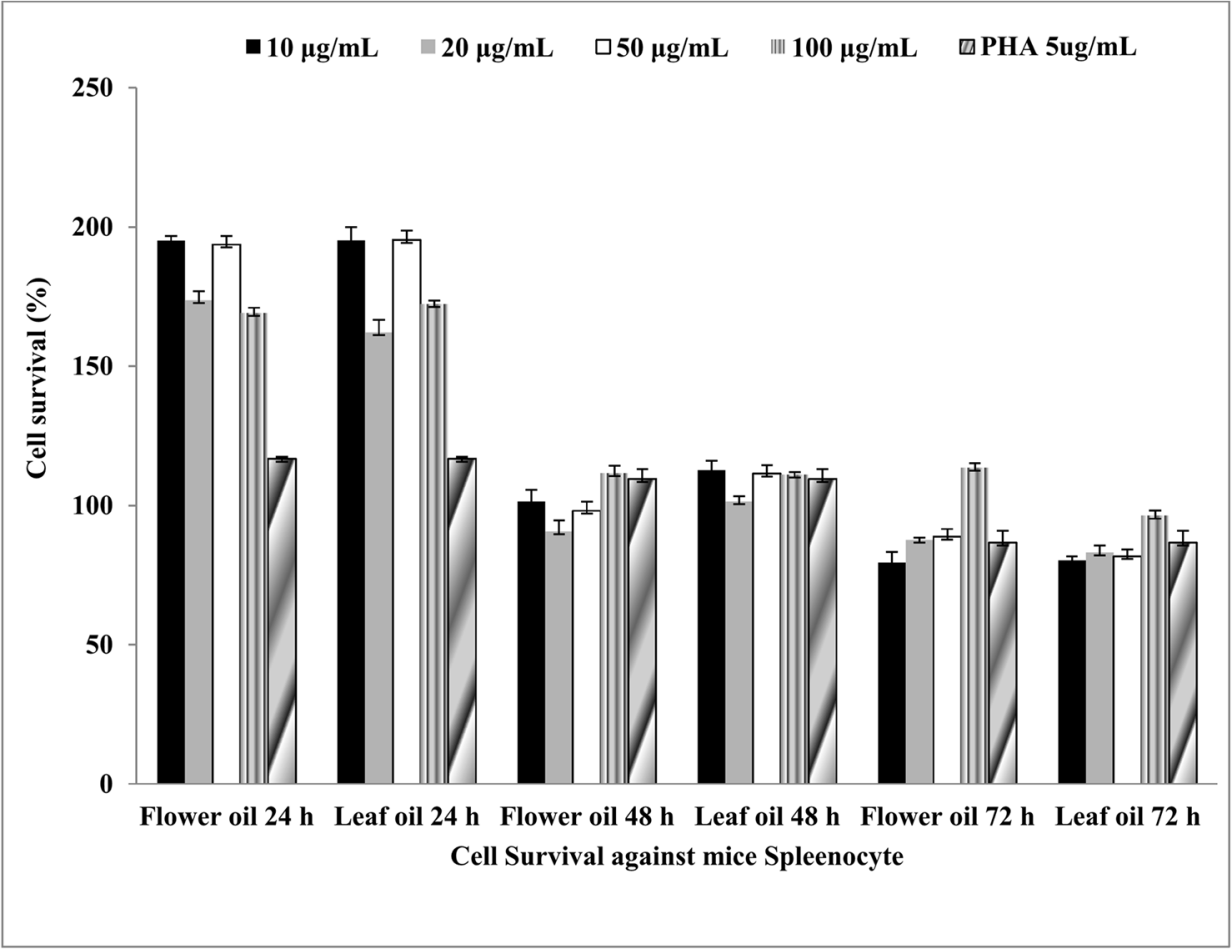
Immunomodulatory activity of leaf and flower oils against mouse splenocytes by SRB assay.

**Fig 6:**
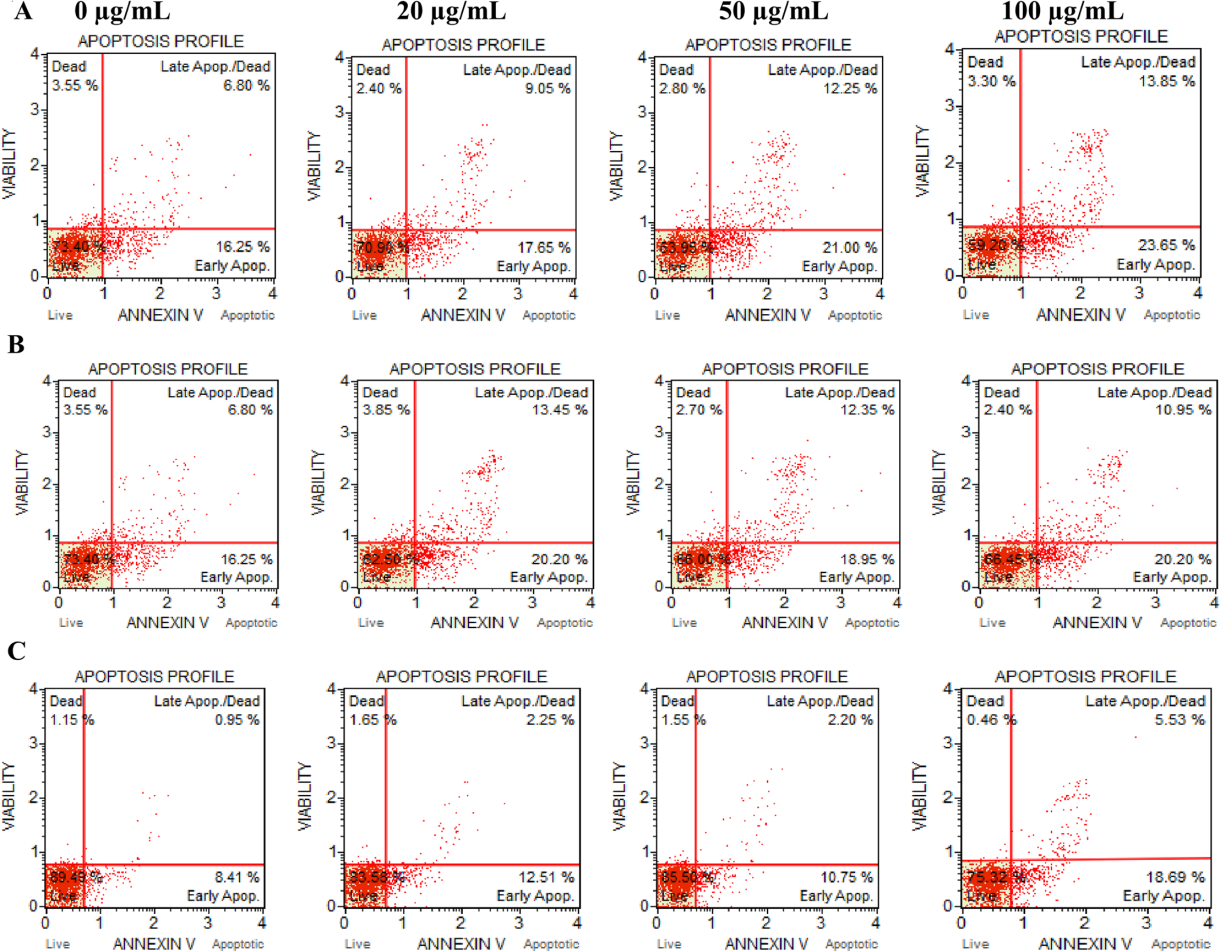
Induction of apoptosis on A549 and C-6 cells by leaf and flower oils at 20, 50 and 100 μg/mL assessed by flow cytometry. (A) A549 cells treated with flower oil for 12 h. (B) A549 cells treated with leaf oils for 12 h. (C) C-6 cells treated with flowers oil for 24 h.

Flower oil (100 μg/mL) showed highest cytotoxic activity against A549 (66.7%±2.2, p<0.001) and C-6 cells (69.1%±3.1, p<0.001) with IC_50_ value of 39.8 to 77.8 μg/mL (Fig 2A). However, it did not show remarkable activity against SiHa and Colo-205 cells. In contrast, 100μg/mL leaf oil showed significant activity against A549 cells (61.4%±5.0, p<0.001) with the IC_50_ value of 84 μg/mL but not against C-6, SiHa and Colo-205 cells (Fig 2B). A concentration dependent cytotoxicity of flower and leaf oils with IC_50_ value in the range 39.8 to >100 μg/mL was recorded (Table 2) but their effects on SiHa and Colo-205 cells were insignificant. However, morphological changes such as reduction in cell number and cellular shrinkage, were observed in the A549 (Fig 3A–3F) and C-6 cells (Fig 3G–3J) treated with increasing concentration nd treatment duration.

**Table 2:** IC_50_ value against A549 & C-6 cells in μg/mL.

Test sample	A549	C-6	Colo-205	SiHa
Flower oil of C. citrinus	39.8	77.8	>100	>100
Leaf oil of C. citrinus	84.0	>100	>100	>100

### Cytotoxicity against human peripheral blood mononuclear cells (PBMCs)

MTT assay was used to evaluate the *ex-vivo* cytotoxicity of flower and leaf oils against human PBMCs at the tested concentrations. The survival of PBMCs was quantified by determining the formation of formazan crystals. The cells supplemented with complete medium were used as negative control, whereas the cells exposed to Vinblastine (1μM) were used as positive control. The flower and leaf oils showed 67.3%±1.7 (p<0.001) and 79.6%±1.5 (p<0.001) cell survival at 100 μg/mL, respectively, thereby suggesting that the tested oils are non-toxic to PBMCs at all tested concentrations when exposed up to 72 h (Fig 4).

### Immunomodulatory activity against mouse splenocytes

SRB assay was used to determine the immunomodulatory activity of *C*. *citrinus* oils on mouse splenocytes. The cells supplemented with complete medium were used as negative control, whereas the cells exposed to Phytohaemagglutinin (PHA) at 5 μg/mL were used as proliferation control. Significantly higher proliferation (95.3%±3.3, p<0.001 and 95.1%±1.6, p<0.001, respectively) was recorded in case of flower and leaf oils at 24 h (Fig 5) as compared to PHA (16.7%±0.7). While 1.5%±3.9 and 11.5%±2.7 proliferation were recorded after treatment with 10 and 100 μg/mL flower oil at 48 h, respectively. Leaf oil elicited proliferation at all concentrations. However after 72 h, 13.4%±1.7 proliferation at 100 μg/mL flower oil but none in case of leaf oil were recorded.

### Apoptosis assay

Marked apoptosis in a dose-dependent manner was recorded in the A549 and C-6 cells treated with 20, 50 and 100 μg/mL leaf and flower oils for 12–24 h (Fig 6). When the levels of active caspase-3/7 were studied (Table 3), higher RFU was recorded in case of 100 μg/mL flower and leaf oils as compared to the vehicle-treated (DMSO) cells. It indicates that both the oils caused anti-prolofirative effect on A549 and C-6 cells by inducing apoptosis.

**Table 3:** Caspase 3/7 activity against A549 and C-6 cells.

Sample	Conc.	Relative Fluorescence Units (RFU)			
	(μg/mL)	Flower oil		Leaf oil	
		A549	C-6	A549	C-6
Flower and leaf	10	8682	7315	3724	2504
oils of *C*. *citrinus*	20	9033	8990	4242	4162
	50	29377	9471	4350	4066
	100	42448	22887	88619	8077
Vehicle treated	1% DMSO	8703	8703	3525	3525

### Caspase 3, 7, 9 and PARP activation

**Fig 7:**
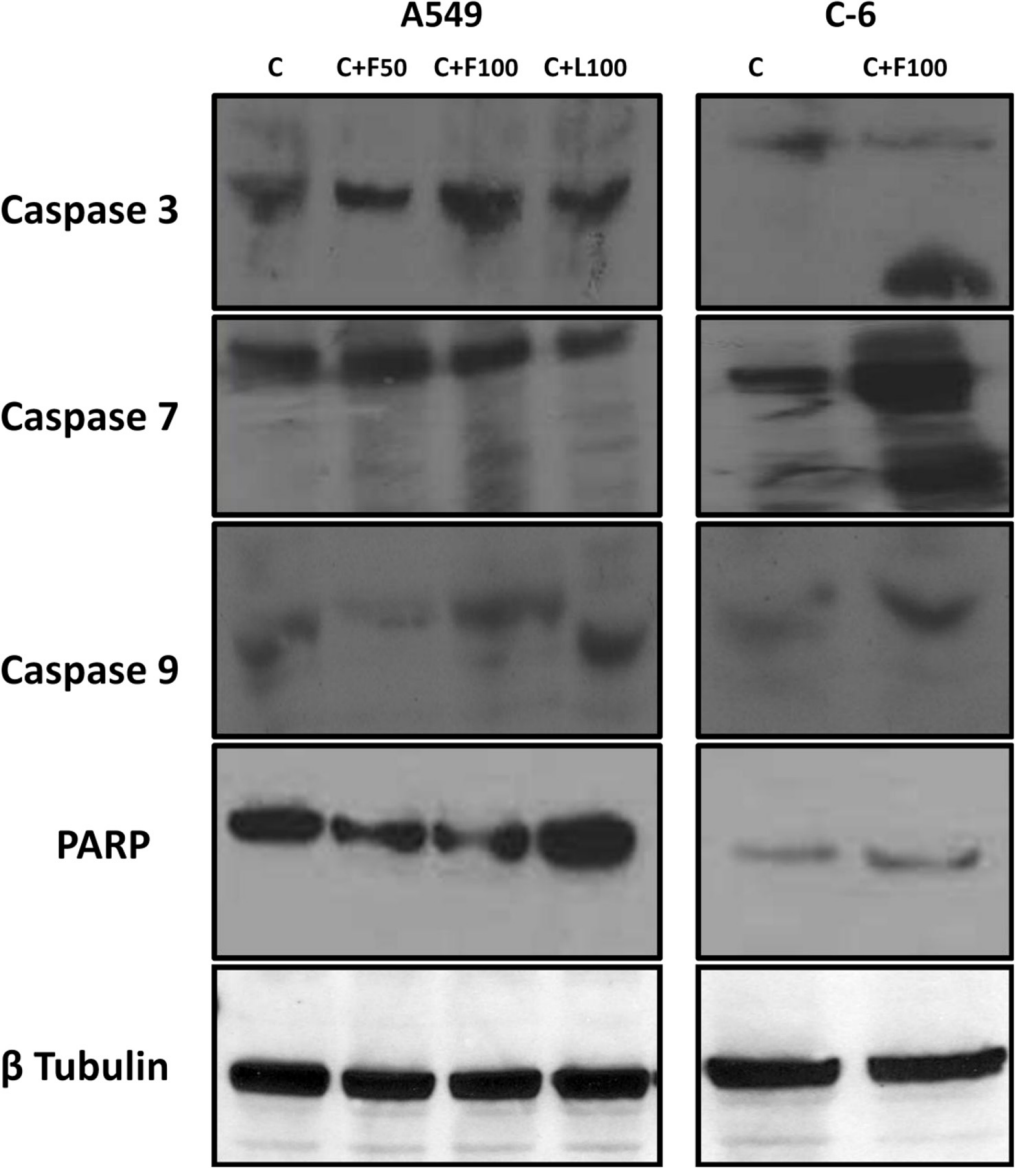
Activation of caspase 3, 7, 9, PARP and β-tubulin by western blot analysis after 36 h treatment with essential oils of (flowers and leaves) *C*. *citrinus*.

When cell lystaes of A549 and C6 cells were fractionated on 10% SDS-polyacrylmide gels and transferred to the PVDF membrane followed by immunoprobing with anti caspase 3, 7, 9 and PARP antibodies, it was found that caspase 3, 7 and 9 are active in both the cell types treated with essential oils of (flowers and leaves) *C*. *citrinus* (Fig 7). Further our observations indicate that essential oil of flowers was not active at 50 μg/ml against A549 cells in case of caspase 3 and 9. This supports caspase 3/7 colorimetric results. Further when the immunoblots were probed with anti-PARP antibody, it did not showed activation in both the cell types treated with essential oils of (flowers and leaves) *C*. *citrinus* at any concentration.

### Cell cycle analysis and CDKs Expression

**Fig 8:**
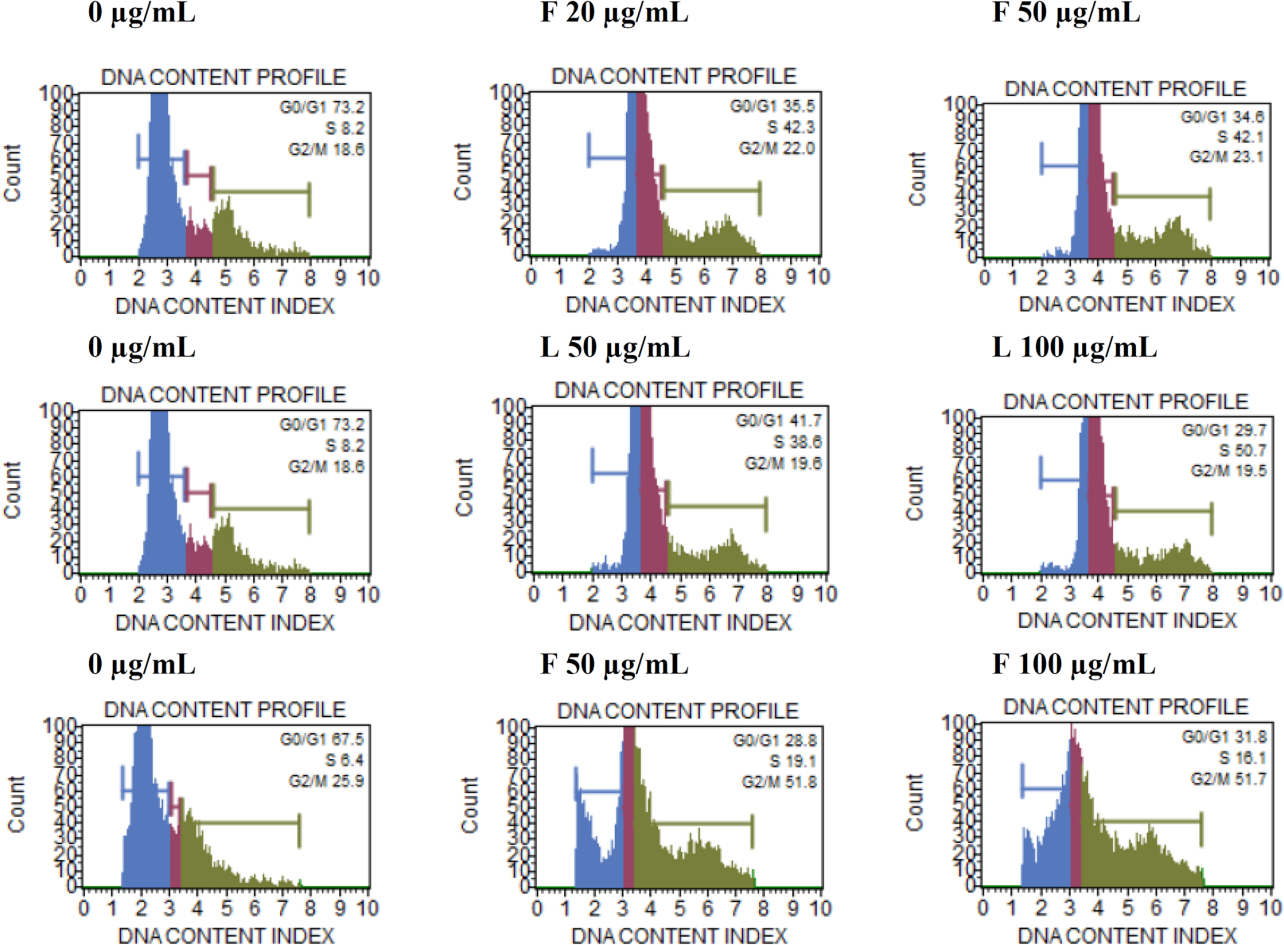
Effect of leaf and flower oils on cell cycle against A549 (A-F) and C-6 (G-I) cells after 24 h. (A) & (D) Control, no treatment. (B) Treated with flower oil at 20 μg/mL. (C) Treated with flower oil at 50 μg/mL. (E) Treated with leaf oil at 50 μg/mL. (F) Treated with leaf oil at 100 μg/mL. (G) Control, no treatment. (H) Treated with flower oil at 50 μg/mL. (I) Treated with flower oil at 100 μg/mL.

Cellular DNA content was analyzed for their cell cycle progression using flow cytometry. After treatment of A549 cells with 20 and 50 μg/mL flower oil, the cells in S phase were almost similar (42.3% and 42.1%, respectively) (Fig 8), whereas, the leaf oil treated A549 cells in S phase increased with increase in concentration (38.6% at 50 μg/mL and 50.7% at 100 μg/mL). However, C-6 cells treated with 50 and 100 μg/mL flower oil were almost same (51.8% and 51.7%, respectively) in G2/M phase.

**Fig 9:**
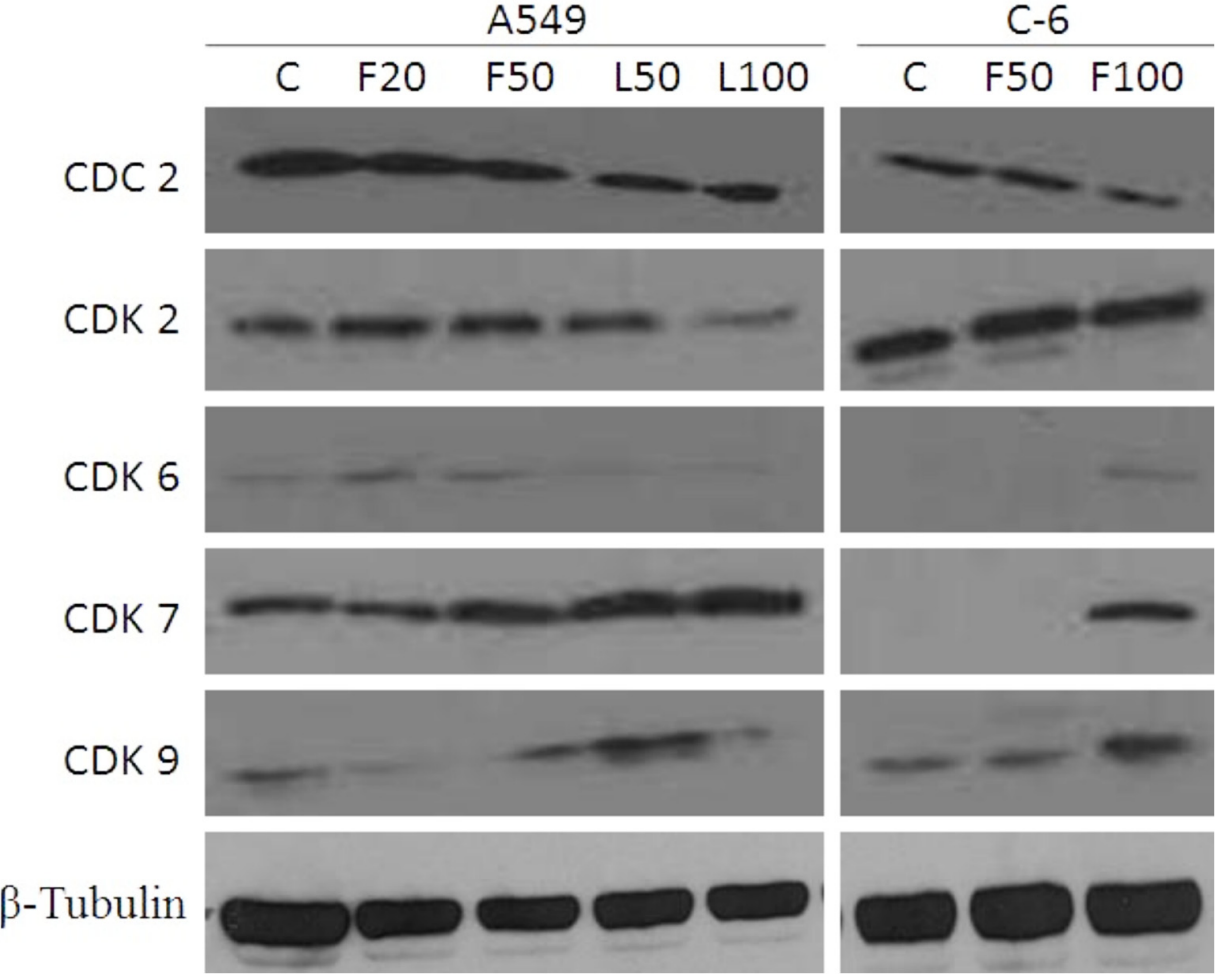
Activation of CDK 2, 6, 7, 9, CDC 2 and β-tubulin by western blot analysis after 36 h treatment with essential oils of (flowers and leaves) *C*. *citrinus*.

In order to further support these findings the lysates of A549 and C-6 cells treated with flower and leaf oils were probed for the expression of different CDKs which includes CDC2, CDK2, 7, 6 and 9. In case of A549 cells, results revealed that CDC2, CDK2 and 7 were prominently expressed in untreated and treated cells. The expression levels of CDC2 were found slightly lesser in case of cells treated with flower and leaf oils than untreated cells whereas, the expression of CDK2 was modulated by the treatment to the lower level. CDK6 expressed with the flower oil treatment at both the concentrations. Expression of CDK7 was observed elevated in cells treated with 50 μg/mL flower oil and at both the concentrations i.e. 50 and 100 μg/mL of leaf oil while CDK9 expression was prominent in cells treated at 50 μg/mL concentration of leaf oil (Fig 9). In case of C-6 cells, CDC2 and CDK2 expression levels were in accordance with the A549 cells. Although there was slight expression of CDK6 in cells treated with flower oil at 100 μg/mL concentration, the levels of CDK7 was prominent in similar set of C-6 cells. Interestingly the expression of CDK9 is higher in the cells treated at 100 μg/mL than that of untreated and the cells treated at 50 μg/mL flower oil.

## Discussion

The conventional treatments of cancer like surgery and chemotherapy pose high risks to the life of patients, and significantly decrease their life span. Therefore, in the recent years, natural and synthetic compounds are being frequently employed for chemoprevention because of their ability to inhibit or reverse carcinogenesis. Natural products serve as antioxidants and anti-proliferative agents and block or suppress the progression of cancer [36].

In view of this, essential oils were obtained from the flowers and leaves of *C*. *citrinus* in the present investigation. The oil yields were higher than that reported by Ming *et al*. [37] but lower than that by Misra *et al*. [38]. Further, the chemical investigations revealed that these oils consists of *α-* and *β*-pinenes, *α-*terpineol, *α-*phellandrene, limonene, *α-*terpinene, linalool, *trans*-pinocarveol, terpinen-4-ol etc. with 1,8-cineole as the major constituent. The results corroborated previous reports [1,16,18–20]. However, major differences between the chemical composition of the leaf and flower oil was recorded in the present study. 1,8-Cineole, *α*-phellandrene, *α-*terpinene, *p*-cymene, isoamyl acetate, isobutyl isobutyrate and in general linear compounds and oxygenated monoterpines were more in the oil of flowers as compared to leaf (Table 1). On the contrary, the leaf oil was dominated by *α*- and *β*-pinenes, limonene, *α-*terpineol and other sesquiterpenoids. Interestingly, the leaf oil contained predominantly higher content of *α*-pinene, limonene and *α*-terpineol as compared to previous studies [18,20,39,40]. It is well known that the variations in the essential oil content and chemical composition are influenced by many factors including but not limited to location, age of the plant, climate, cultivar, method of distillation, type of distillation apparatus employed [41,42]. It is also known that cineole inhibits the growth of leukemia cells, possesses non-reactive and non-toxic properties [43] and also antimicrobial [44], antioxidant [45], anti-inflammatory [46–48] and nematicidal activities [8].

Cytochrome C, an apoptogenic mitochondrial protein released by mitochondrial permeabilization, induces the apoptosis which is the most potent defense mechanism against cancer cell proliferation [49–51]. In the present study, the essential oils exhibited the induction of apoptosis in human lung carcinoma and rat glioma cells (Fig 6). The flower oil demonstrated highest cytotoxicity on A549 and C-6 cells at 100 μg/mL (Fig 2A), whereas the leaf oil exhibited highest activity against A549 and C-6 cells at 100 μg/mL and 50 μg/mL respectively (Fig 2B). However, these oils had insignificant effects on Colo-205 and SiHa cells. Further, A549 and C-6 cells were arrested in S and G2/M phases when they were analyzed for their cell cycle progression (Fig 8). This was also further supported by our observations about the expression levels of CDC2 [52] and CDK2 [53] in case of treated cells with flower and leaf oils. Whereas CDK6 expressed prominently only in case of flower oil at higher concentration owing to its role in cell differentiation and as a regulator of G1 phase of cell cycle which is significant in case of stem cell like C-6 cells [54]. CDK7 expression was observed elevated in A549 cells while in case of C-6 expression was observed only at 100 μg/mL which is significant finding supporting our preliminary observation of cellular arrests in S phase. Expression of CDK9 was prominent in C-6 cells but in A549 cells expression was modulated in cells treated with flower oils at both the concentration and elevated when treated with leaf oil only which indicates some novel trend owing to its constitutive nature of expression in throughout the cell cycle [55] (Fig 9). In addition, the activation of caspase-3/7 expressed in RFUs was found to increase with the concentration of both the oils (Table 3). It suggested that both the oils induced apoptotic cell death through caspase-3/7 dependent processing which is also further confirmed by our western blot results. Further, the percentage of apoptotic cells increased with the concentration. When we probed the cell lysates obtained from the cells treated with essential oils isolated from flowers and leaves *C*. *citrinus* for the expression and activation of the various caspases, including caspase 3, 7, 9 and PARP, it has been found that caspase 3 and 7 gets activated and showed the cleavage in both the cells (Fig 7). However, we did not notice the activation of PARP in both the cells at any concentrations. But caspase 9 showed the promising activation in both the cell types at higher concentration. The inhibition in cell survival was due to apoptosis. Interestingly, both the oils supported the proliferation of mouse splenocytes (Fig 5) and also protect human PBMCs (Fig 4) without causing toxicity.

Present study concludes that the highly active phytochemicals like 1,8-cineole, and monoterpenoids in the essential oils extracted from *C*. *citrinus* flowers and leaves may serve as potent natural anti-cancer compounds with important roles in human health. The flower oil showed promising activity on A549 and C-6 cells but that of leaf oil showed activity on A549 cells only. Cytotoxicity tests at higher concentrations against human PBMCs and mouse primary splenocytes suggested the non toxic nature of the flower and leaf oils *ex-vivo*.
